# Organizations, Present and Future

**Published:** 1902-10

**Authors:** 


### Editorial.

### ORGANIZATIONS, PRESENT AND FUTURE.

In the original department of this number will be found an interesting paper by Dr. William H. Trueman, on the "British Dental Association." It deserves careful reading by all those interested in national, State, and local dental societies, for it shows what a determined and united effort has accomplished for the good of the dental profession in Great Britain.

The contrast between this and our own poorly systematized management tends to reduce the egotistic idea that the average American can do things generally a trifle better than his cousin over the ocean. In the matter of organization the said American is yet, apparently, in his infancy, and the maturity of experience seems far away if we may judge by results and the indifference to the subject manifested throughout dental circles.

While it is true, as Dr. Trueman states, that geographical limitations may have much to do with the solidarity and progress of the dental fraternity in Great Britain, it certainly has not all to do with the great results attained. There must be something in the men themselves worthy as an example for us to follow. The question of training has, doubtless, much to do with it,—training as a class. This latter feeling is so sharply pronounced in England that it leads necessarily to a closer bond of union than could possibly exist in this country, where class distinctions have very little, if any, force. It is this individuality, every one for himself, that continually antagonizes effort to effect a professional unity in the United States. It is, perhaps, not desirable that this condition should be changed, for although class distinctions have an advantage in some directions, they are a bar to individual freedom, and to that extent make a general advancement along any line of work difficult.

With this national characteristic working against unity, it would seem impossible, upon a hasty view, to develop anything better out of the materials and conditions at hand; yet the situation is not entirely hopeless. Those who have watched carefully the changes in the dental profession in the United States during the past fifty years can but feel that while we cannot reach our English brethren in unity of effort, we, with our larger domain and more extended difficulties, have made good progress towards this most desirable condition.

The question of organization has become a problem, and one requiring the best thought of serious minds. The solution is not one to be quickly thought out or quickly put in practice. The mental peculiarities must undergo a partial and radical change before anything of moment can be developed. It may be said, if this be so, why attempt any improvement? The answer must be that education alone can effect the result. All means should be resorted to to bring the intellectual forces of the American mind to see the importance of this subject, and while it may be long developing, there will come a time when the warped mentality will assume more perfect proportions.

To accomplish this strenuous efforts should be made use of to effect results. The literature of the dental profession must be established upon a true professional basis. It must be above and beyond commercial temptations and environment. It must be above all reproach, and hold the standard higher than those do who are supposed to be trained by it. It must lead, and never descend from its high estate to gratify degrading impulses. This is a high mission. Is it being fulfilled by the periodical literature of dentistry in America? The answer cannot be made here, but the fact remains that the great body of the twenty-five thousand dentists in the United States have not reached a standard of unity to be compared with that of England, Ireland, and Scotland, notwithstanding the fact that the organization of dental societies and colleges had their origin here. Who is mainly responsible for this? It seems to the writer that the solution of this problem constitutes the most important question now devolving upon the dentists of this country. Are we prepared to grasp it and meet its difficulties?

An important factor that has been a permanent bar to progress has been our methods of organization. This applies with variable force to all, local, State, and national societies, but especially so to the latter. What conclusion must be reached when it is remembered that from two to three hundred members of this body meet annually under the name of the National Dental Association, and this out of twenty-five thousand dentists in the country? There can be but one answer, and that is that the body is non-representative and cannot possibly dominate the professional thought of the great mass. It is a serious question whether it has any marked influence for good in any direction. While numbers are not always important, for one may oftentimes "chase a thousand," still the small number that meet once a year, while good so far as it goes, indicates a state of mental torpor at large in dentistry that is anything but encouraging.

The American Dental Association and its successor, the National Dental Association, have each been founded upon a standard not higher than the level of mind they are supposed to represent. This has been from the beginning the fatal mistake. The teacher must be in advance of the pupil, the leader, of his followers. The National Association has followed the lead of the American and degenerated into a clique of permanent members. It has ceased to be representative, and while the farce is annually gone through with of electing representatives in State and local societies, these bodies are never informed officially of the proceedings of the superior body, nor are they permitted to have a bound copy of its proceedings. These are not to be secured by money and certainly never by love. The natural result is that no unity exists between these organizations. The National Association is to the average dental mind something apart and divorced from his immediate professional life. It meets once a year, and the members enjoy the reunion, but the effect upon the profession for good is an infinitesimal quantity; few know of its work and fewer still read its proceedings. If the plan of selling these to the highest bidder, and that to the exclusion of all other dental journals is, as the writer understands, now in force, the national body might with propriety resolve itself into a dental fraternity, with the usual initiation ceremony, grip, and passwords, for it will have ceased to be an open factor for good.

The association of the future, whenever formed, should combine liberality with conservatism, a not impossible combination. It should be representative in character, but representation should be based on recognized ability. Permanent membership should have no place in this body, nor would it be necessary. It should be so intimately related to subordinate associations that the latter would feel it to be a part of their professional possessions. The subordinate bodies should financially aid the superior organization in proportion to their representation, and in that proportion should be entitled to copies of the proceedings. Details of such an organization cannot be worked up here, but it must be apparent that the present methods are a dead weight and can never lead to harmony of effort or to a standard of excellence worthy the efforts and individual progress made in this country.

The future promises two methods of national organization. One in which there should be a liberal representation in the national body. Instead of two or three hundred, there should be five thousand members. This would give an income sufficient to publish the proceedings and avoid the disgraceful method now prevailing. It should have a dental home, possibly in Washington, D. C., with a library worthy the name and with all the conveniences for reference and original research. The main body could, as now, meet East, West, and South, and perform the missionary work of interesting outlying and isolated sections. There seems no reason why, with good business methods, an income of fifty thousand dollars could not be secured. It means work, however, and intelligent methods. This is but the extension of older systems of organization in this and other countries.

There might be organized another, also representative, but the men and women who represent it should be adepts in original investigation. These should meet in national convention and lead the dental profession through the evidence of accomplished work. Such a body would be regarded with a respect not at present given to national organizations, for it would come to be recognized as the final arbiter on disputed questions.

That such an association will be formed in the near future is not probable, but the enlargement of the scope of work through present systems seems not only probable, but very possible of attainment, and its accomplishment should be considered as one of the important things in the next decade.

It is believed the time is nearly ripe for an organization representing all the best local societies of the country, and upon a basis that will satisfy the progressive ideas of the present century. To meet this want the work must cover the entire product of dentistry. Nothing too low or too high to be omitted. There is no organization now existing that meets this demand. Is there sufficient energy among the dentists of the country either to modify those now existing, or, if that be not possible, to organize something that will meet all the conditions and elevate our calling to a place worthy the age and its recognized value as one of the beneficent professions?

